# Non-absorbable Suture-Loaded Anchors for Finger Pulley Reconstruction

**DOI:** 10.7759/cureus.61250

**Published:** 2024-05-28

**Authors:** Francisco Rodriguez Fontan, Renzo Laynes, Frank Scott

**Affiliations:** 1 Faculty of Medicine, Universidad de Buenos Aires, Buenos Aires, ARG; 2 Department of Orthopedics, University of Colorado Anschutz Medical Campus, Aurora, USA

**Keywords:** trigger finger release, suture tape, pulley reconstruction, flexion contracture, bowstringing

## Abstract

A 59-year-old woman, who previously underwent surgery on her left long finger A1 pulley and left small finger distal interphalangeal joint for triggering and mallet deformity at another medical facility in March 2021, sought evaluation at an Orthopedics Hand clinic. She presented with limited finger movement, a flexion contracture, and difficulty extending her left long finger. Examination revealed an A2 pulley injury with extensive scar tissue. Subsequently, she underwent surgery to remove the scar tissue and reconstruct the A2 pulley using suture tape anchors. This case highlights the negative outcome following A1 pulley release due to an unintended A2 injury, resulting in significant scarring and an intrinsic plus digit posture. Additionally, it underscores the potential effectiveness of using non-absorbable synthetic sutures to minimize scarring and promote an early range of motion in cases where healing leads to excessive scarring around the flexor tendon sheath.

## Introduction

The pulley system is a fibro-osseous structure that allows biomechanical tendon gliding efficiency and nutrition [1]. There are open and closed pulley injuries. Pulleys A2-4 injuries should be reconstructed to avoid bowstringing and flexion lag [1-3]. The open ones are usually seen in the setting of lacerations and crush injuries but can also be iatrogenic when performing trigger finger release [1-5], whereas the closed ones are seen with eccentric or strenuous loading to fingers such as rock climbing and baseball pitchers [1,6]. This case presents an inadvertent iatrogenic A2 pulley injury during trigger finger release [5]. Notably, the A1 and A2 pulleys edges can be occasionally challenging to determine due to anatomical continuity (~40% cases) or inflammation [2,5]. Bowstringing after trigger finger release can be seen when associated A2 release beyond its 25% is compromised [2,5].

This article briefly reviews pulley reconstruction options, in association with a case of a pulley graft-free reconstruction with suture tape anchors as a substitute. The patient provided written consent after she was informed that data concerning the case could be submitted for research and publication.

## Case presentation

This is a 59-year-old, otherwise healthy right-hand dominant female, who had a left long finger (LF) A1 pulley release and a left small finger (SF) distal interphalangeal (DIP) joint pinning for triggering and mallet deformity, respectively, at an outside institution in March 2021. She presented to the Orthopedics Hand clinic for evaluation three months post-intervention with a chief complaint of persistent left LF pain with a lack of full extension and flexion, and grip weakness. Post-operatively, she had the SF pin removed in April and had been treated with extension splinting for both SF and LF as well as scheduled hand therapy sessions.

**Figure 1:**
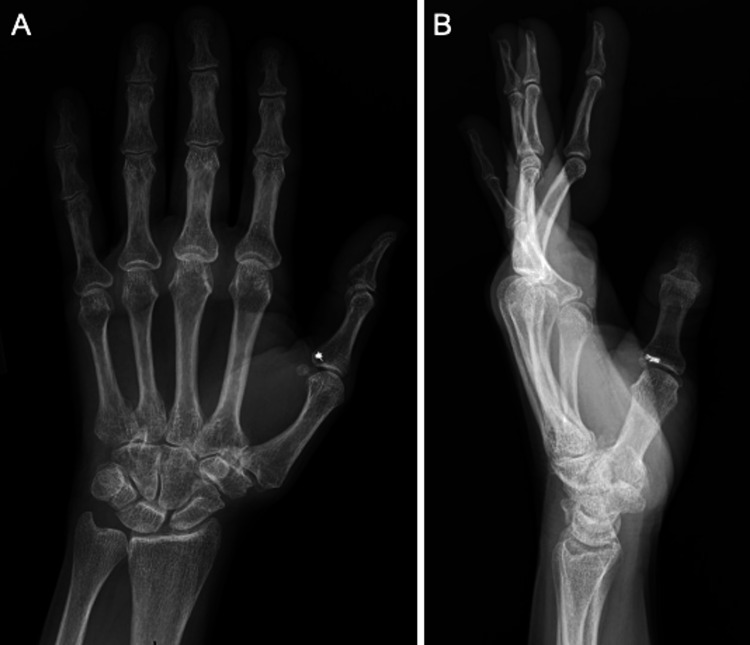
Posteroanterior and lateral radiographs of the hand (A and B) posteroanterior and lateral x-ray views of the left hand. On attempting extension, note extension lag on the lateral view of the long finger at the metacarpophalangeal joint, with hyperextension of the proximal interphalangeal joint. There was no significant long finger interphalangeal osteoarthritis. Thumb with retained hardware from remote ulnocollateral metacarpophalangeal ligament repair.

It was noted on the left LF that she had significant scarring surrounding the volar aspect at the site of the A1 pulley release, with a palpable central cord-like structure. The LF had a 35-degree metacarpophalangeal (MCP) joint flexion contracture. It could be passively corrected to a 10-degree extension lag. The proximal interphalangeal (PIP) joint was held at rest in hyperextension manifesting as early swan neck deformity as it had partial active and passive flexion. Bunnell’s test was positive for differential intrinsic tightness (ulnar > radial) with postural ulnar LF deviation and flexible PIP joint. This was noted when the patient was unable to make a composite fist with flexion of the LF. Of note, she was experiencing paresthesias and numbness along the radial and ulnar LF borders and the radial ring finger (RF) border. Given the inability to extend the LF MCP joint with flexor tendon bowstringing and the associated intrinsic plus contracture deformity (i.e., MCP joint flexion contracture and PIP joint extension) noted during the visit, it was determined that she likely had an A2 pulley injury (Figure 1). She was also noted to have an SF DIP 10-degree extension lag that could be passively corrected, but this was not as problematic for the patient compared to the LF findings and seemed to be improving with extension splinting.

 A corticosteroid injection in the region of her LF A1 pulley for scar softening and serial dynamic custom-fitted extension splinting was attempted. She followed up closely with no significant improvement. At her three-month follow-up, the left LF range of motion (ROM) had regressed to approximately the same MCP 40-degree flexion contracture and did not passively correct as before. Given failed non-operative management, surgery was recommended. The planned surgery was contracture release with an exploration of the tendon and possible pulley reconstruction. Due to her history of significant scarring, and to reduce the morbidity of using autograft tendon, a less invasive procedure was deemed to be in her favor. Reconstruction using only suture tape-loaded anchors (Arthrex; Naples, FL, USA) was determined to be the most seemingly applicable option. 

Operative technique

The procedure was performed under general anesthesia in the supine position. A Bruner’s-like incision was made over the volar aspect of the LF MCP including the prior surgical scar at the distal palmar crease (DPC) and extending over flexor zones 2-3. A significant pretendinous longitudinal fibrosed and thickened tissue overlying and attached to the flexor tendons were identified. The digital neurovascular bundles, radial and ulnar, were traced proximally where normal appearing anatomy was discernible. Distally, the neurovascular bundles were found tethered and encased in fibrotic tissue, requiring extensive sharp neurolysis to free the nerves and vessels. The longitudinal scarred fibrotic tissue was excised proximal to distal. Significant scarring involving the MCP volar plate, distal tendinous lumbrical, and interossei extensions were noted deep to the flexor tendons. The MCP capsulotomy and volar plate release with fibrous tissue excision followed, in addition to tenotomy of the lumbrical and involved interossei distal to the MCP joint. At this point, the contracture and intrinsic tightness improved significantly and the finger resting posture was passively correctible. 

**Figure 2:**
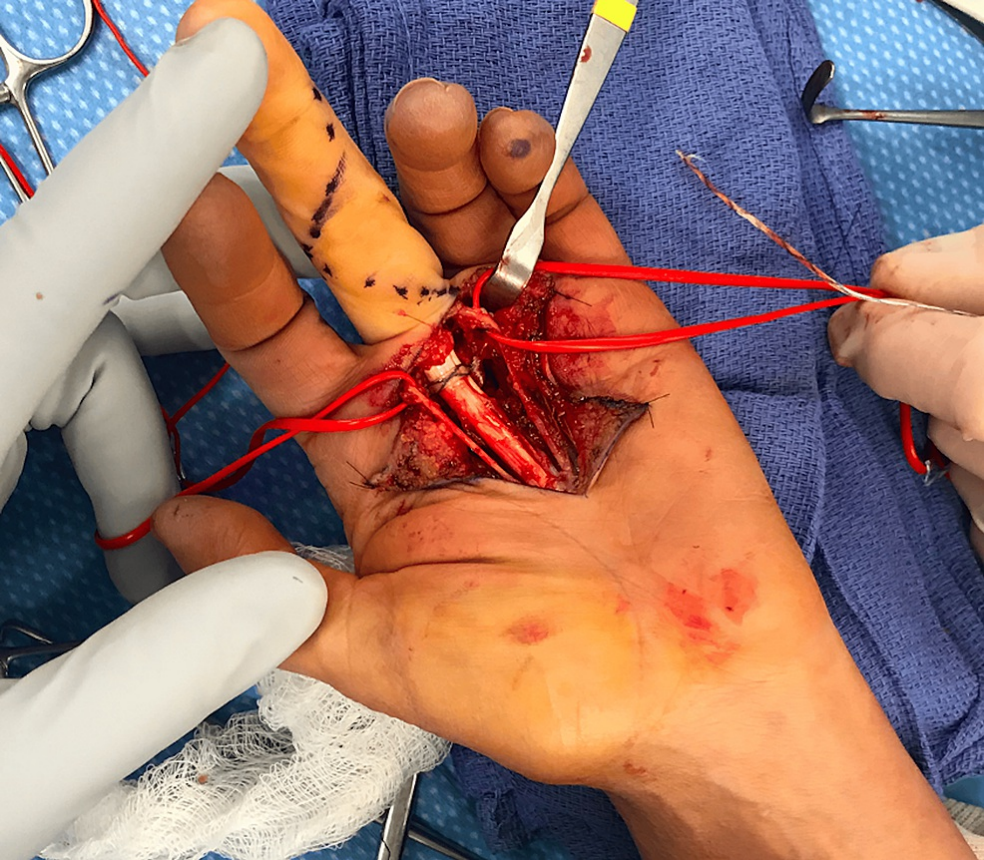
Intraoperative image The intraoperative image shows the position of the first suture tape overlying the flexor tendons of the long finger at the proximal phalanx. Red vessel loops are gently retracting and holding neurovascular bundles.

For the A2 pulley reconstruction, the base of the LF proximal phalanx was exposed radially and ulnarly for positioning anchors less than 1 cm distal to the MCP joint. After pre-drilling with the guidewire, the most proximal and radial single-loaded all-suture tape loaded anchor was placed first (FiberTak DX Suture Anchor, 1.3 mm Suture tape). Next, an unloaded second suture anchor (Mini Suture Anchor, 2.5 x 8 mm BioComposite PushLock) was placed on the ulnar side and at the same level, passing the two strands of the suture tape from the first anchor through the eyelet prior to impaction. Just parallel, and slightly distal to these anchors, another pair of PushLocks anchors were used by positioning first the preloaded radial anchor with 1.3 mm suture tape, and next the ulnar anchor by passing the two strands of the suture as mentioned. Adequate tension was obtained with low profile knotless technique while impacting the suture anchors during passive flexion and extension testing (Figure 2 and Video 1). The ulnar flexor digitorum superficialis (FDS) tendon slip was excised at Camper’s chiasm through a small additional incision on the volar aspect of the PIP. There was adequate passive excursion without triggering in the radial FDS and flexor digitorum profundus. Standard wound closure was followed with interrupted 5-0 prolene suture and a volar resting splint was placed.

**Video 1:** Pulley reconstruction Pulley reconstruction with suture tape-loaded anchors.

At two weeks post-operatively, the incision was healing without complications. Sutures were removed and was transitioned to a custom-fitted dorsal LF MCP/PIP static progressive extension daytime use orthosis, except for ROM exercises. On exam, there was diffuse digital swelling and active limited DIP and PIP flexion. The PIP had flexible 30-degree flexion contracture, but no postural MCP flexion contracture with active minimal flexion and extension. The sensation was intact over the radial border of the RF but was decreased over the radial and ulnar borders of the LF distal to the incision. There was a positive Tinel’s sign over the radial and ulnar aspect of the proximal incision. She began ROM precautions with active/passive PIP/DIP motion with MCP in extension, MCP passive extension only. 

At one month, the incisions continued to heal uneventfully. ROM significantly improved to being able to actively flex more the DIP/PIP/MCP joints and had a 4 cm lag from DPC on full active LF flexion. Overall, active ROM was: 0-20-degrees MCP, 30-85-degrees PIP, and 10-70-degrees DIP. The sensation continued to be decreased over the radial and ulnar borders distal to the incision (>10mm 2-point discrimination). Splinting indications were transitioned to use intermittently and to begin progressive gentle use of the hand with ROM as tolerated and 5-pound weight restriction. 

At two months, incisions healed well with some raised skin areas. There was no evidence of flexor tendon tenderness. The ROM continued to improve and had a 2 cm lag from DPC. Overall, active ROM was: 0-75-degrees MCP, 20-90-degrees PIP, and 0-70-degrees DIP. The LF was noted slightly ulnar deviated and had some ulnar intrinsic tightness. The sensation continued to be decreased but improved since the last visit. At this point, she was encouraged to begin resistance exercises and minimize splint use throughout the day. 

**Figure 3:**
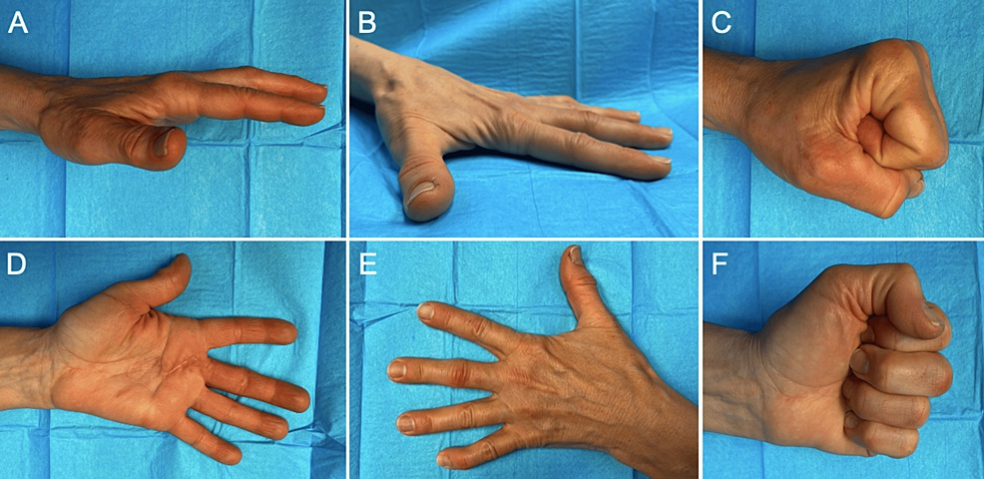
Six-month follow up (A-F) Full digits range of motion from resting posture (A, D, E), and active extension (B) to preforming a full composite fist (C, F) from different views.

At six months, her function had significantly improved. She was engaging in more activities such as gardening and skiing. She could make a full composite fist, and the LF had mild ulnar drift and differential ulnar intrinsic tightness with a PIP 10-degrees active extension lag (Figure 3 and Video 2). She was reporting mild paresthesias over the LF pulp only. At this point, the patient continued night time extension splinting and regular exercises focusing on PIP extension. 

**Video 2:** Six-month follow-up Able to make a full fist at six months.

**Figure 4:**
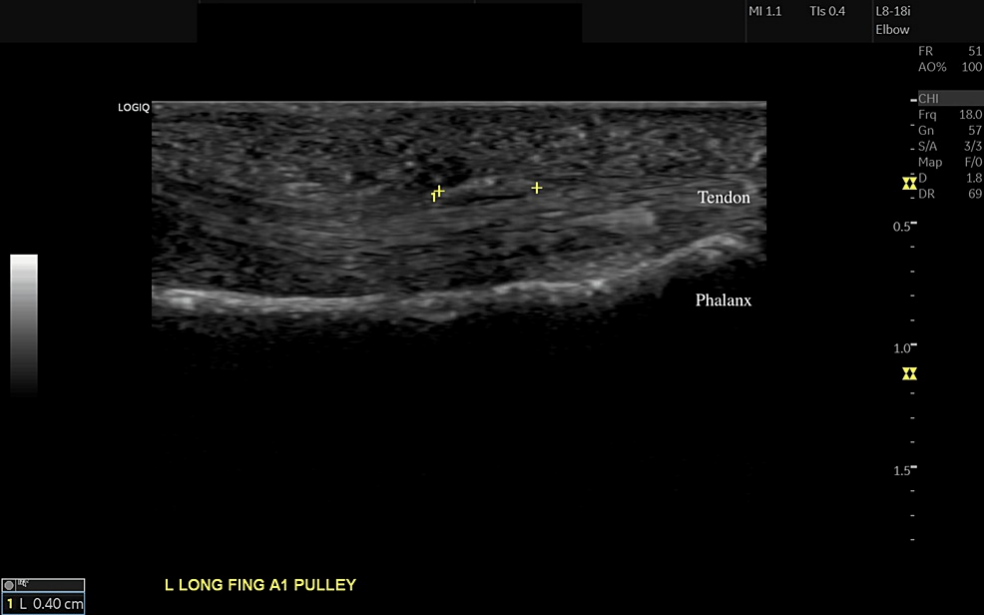
Ultrasound at 1.5 years follow up Ultrasound of the left long finger longitudinal axis. Between yellow crosses is the suture tape.

At 1.5 years, she continued to have the same ROM and had no discomfort on the LF. The PIP had approximately 20-degrees active extension lag. Regarding the paresthesias this had resolved and had normal sensation to light touch around the digit (Video 3). An ultrasound was performed in a clinic demonstrating normal gliding of the tendons with no signs of inflammation, no fraying, nor degeneration and intact suture fibers (Figure 4, and Videos 4-5). A static dorsal PIP extension splint for night time use was provided to improve terminal extension.

**Video 3:** Follow-up at 1.5 years Able to make a full fist at 1.5 years.

**Video 4:** Ultrasound Ultrasound of the tendon in longitudinal axis.

**Video 5:** Ultrasound Ultrasound of the tendon in axial axis.

## Discussion

Surgical repair is not sufficient to withstand the biomechanical strain the pulley needs [6]. Among the described techniques are the utilization of the remnant pulley rim and “looping” [3]. The utilization of a graft through the rim in a “shoelace” or “interweave” fashion is biomechanically weaker and failure is usually seen at the graft-rim interface [3]. The looping option has many variants. The “belt loop” is essentially passaging the tendon under the volar plate. This method is advantageous for associated flexor tendon injuries, staged reconstructions, and when reconstructing A1-3-5 pulleys; however, it is stiffer, increases the friction and work of flexion, and may lead to joint erosion [3]. The “single-, double-, loop-and-a-half, or three-loop” involve similar techniques implicating the use of a tendon graft to be circumferentially passed around the phalanx [3,7]. The three-loop is superior in replicating the native pulley in strength and length, and more efficient at preventing bowstringing [7]. A hybrid technique tested in cadavers, involving a “half loop” palmaris longus graft attached to the bone with two fiber wire suture-loaded anchors, has found similar strength to single-loop but was significantly weaker to double-loop [7]. Other investigated options include allografts, xenografts, acellular dermal matrix, and synthetics [8]. Some of these materials are not strong enough and elicit foreign body reactions and adhesions [8]. 

In this case report, the use of an all synthetic-based construct over a gliding tendon can raise concerns for possible flexor tendon attritional rupture or inflammation. However, prior sports literature found grafts and sutures to undergo synovialization [9], which makes us consider that this phenomenon could be extrapolated to this case. However, this assumption warrants further investigation. 

To our knowledge, there are prior reported cases of A2 pulley injury after trigger finger release [5], but no prior case showing graft-free pulley reconstruction with knotless suture tape anchors. This case describes the outcome of a patient who had A2 pulley reconstruction using suture tape as a synthetic substitute. In our case, there was no viable remnant pulley rim, and the significant fibrosis of the flexor tendons was not a suitable option for local harvest or use, and preference over a minimally invasive technique with a lower profile was advocated for knotless suture tape-based anchors [10].

## Conclusions

In conclusion, this case report highlights the successful outcome of A2 pulley reconstruction using knotless suture tape anchors as a synthetic substitute in a patient with no viable remnant pulley rim and significant fibrosis of the flexor tendons. While prior cases have documented A2 pulley injuries post-trigger finger release, our approach stands out for its novel use of the graft-free pulley reconstruction technique. Our preference for this minimally invasive technique underscores its advantages in achieving favorable outcomes, emphasizing the importance of innovative approaches in hand surgery for improved patient care and functional outcomes. Further studies and long-term follow-ups are warranted to validate the efficacy and durability of this approach.
